# Identifying neuropsychiatric disorders in the Medicare Current Beneficiary Survey: the benefits of combining health survey and claims data

**DOI:** 10.1186/s12913-016-1774-y

**Published:** 2016-10-01

**Authors:** Sophia Miryam Schüssler-Fiorenza Rose, Dawei Xie, Joel E. Streim, Qiang Pan, Pui L. Kwong, Margaret G. Stineman

**Affiliations:** 1 Spinal Cord Injury Service, Veterans Affairs Palo Alto Health Care System, 3801 Miranda Ave (MC 140), Palo Alto, CA 94304 USA; 2 Department of Neurosurgery, Stanford University, Stanford, California USA; 3 Department of Biostatistics and Epidemiology, The Center for Clinical Epidemiology and Biostatistics, Perelman School of Medicine, University of Pennsylvania, 423 Guardian Drive, Blockley Hall, Philadelphia, PA 19104-6021 USA; 4 Geriatric Psychiatry Section, Department of Psychiatry, Perelman School of Medicine, University of Pennsylvania, Philadelphia, Pennsylvania 19104 USA; 5 VISN 4 Mental Illness Research Education and Clinical Center, Corporal Michael J. Crescenz Veterans Affairs Medical Center, Philadelphia, Pennsylvania USA; 6 Department of Physical Medicine and Rehabilitation, Perelman School of Medicine, University of Pennsylvania, Philadelphia, Pennsylvania 19104 USA

**Keywords:** Administrative claims, Health surveys, Dementia, Intellectual disability, Developmental disabilities, Central nervous system diseases, Mental disorders, Medicare Current Beneficiary Survey, MCBS, Residential facilities

## Abstract

**Background:**

To address the impact of using multiple sources of data in the United States Medicare Current Beneficiary Survey (MCBS) compared to using only one source of data to identify those with neuropsychiatric diagnoses.

**Methods:**

Our data source was the 2010 MCBS with associated Medicare claims files (*N* = 14, 672 beneficiaries). The MCBS uses a stratified multistage probability sample design to select a nationally representative sample of Medicare beneficiaries. We excluded those participants in Medicare Health Maintenance Organizations (*n* = 3894) and performed a cross-sectional analysis. We classified neuropsychiatric conditions according to four broad categories: intellectual/developmental disorders, neurological conditions affecting the central nervous system (Neuro-CNS), dementia, and psychiatric conditions. To account for different baseline prevalence differences of the categories we calculated the relative increase in prevalence that occurred from adding information from claims in addition to the absolute increase to allow comparison among categories.

**Results:**

The estimated proportion of the sample with neuropsychiatric disorders increased to 50.0 (both sources) compared to 38.9 (health survey only) and 33.2 (claims only) with an overlap between sources of only 44.1 %. Augmenting health survey data with claims led to an increase in estimated percentage of intellectual/developmental disorders, psychiatric disorders, Neuro-CNS disorders and dementia of 1.3, 5.9, 11.5 and 3.8 respectively. In the community sample, the largest relative increases were seen for dementia (147.6 %) and Neuro-CNS disorders (87.4 %). With the exception of dementia, larger relative increases were seen in the facility sample with the greatest being for intellectual/developmental disorders (121.5 %) and Neuro-CNS disorders (93.8 %).

**Conclusions:**

The magnitude of potentially underestimated sample proportions using health survey only data varied strikingly according to the category of diagnosis and setting. Augmentation of survey data with claims appears essential particularly when attempting to estimate proportion of the sample affected by conditions that cause cognitive impairment which may affect ability to self-report. Augmenting proxy survey data with claims data also appears to be essential when ascertaining proportion of the facility-dwelling sample affected by neuropsychiatric disorders.

## Background

The United States Medicare or “Health Insurance for the Aged and Disabled” program provides coverage for almost 44 million Americans ages 65 and older and 9 million Americans with a long-term disability [1]. People with neuropsychiatric impairments comprise a substantial portion of Medicare beneficiaries, but obtaining accurate prevalence estimates can be challenging. Those with cognitive impairment may under-report conditions associated with cognitive impairment due to lack of insight, stigma and other factors. Surveys may only ask about a limited number of conditions. Similarly, administrative data can have low sensitivity [2] and may miss cases. Administrative data, however, are commonly used in health services research because of its availability. Studies involving dementia patients have shown that self-report, administrative data and other sources are complementary; to obtain accurate prevalence estimates one should use multiple sources of data [3]. However, dementia is only one of several health conditions that can account for cognitive and other neuropsychiatric impairments in the Medicare population. In particular, beneficiaries with intellectual disabilities and those with severe mental illness or dementia comprise a substantial portion of the under-65 disabled Medicare population (37 %) [4]. Since on average they are much younger (62 and 50 % of those with intellectual disability and severe mental illness are under age 45), they tend to be on Medicare longer and can incur substantial Medicare expenditures [4].

There is increasing recognition of the need to improve public health surveillance of people with intellectual and developmental disabilities (ID/DD), with calls for improving data systems and sources from the US Surgeon General in 2002 and 2005 and the Centers for Disease Control and Prevention (CDC) in 2009. One approach to improving public health surveillance is to improve the use of existing data through novel analytic methods [5]. In addition to ID/DD and dementia, other conditions such as severe mental illness, stroke and neurological disorders affecting the brain may lead to cognitive and other neuropsychiatric impairments. These impairments are among the most disabling and persistent. They affect substantial proportions of people who qualify for Social Security Disability Insurance and subsequently for Medicare.

The Medicare Current Beneficiary Survey (MCBS) is an ongoing survey of Medicare beneficiaries which combines multiple sources of data, including Medicare claims and survey data collected from a representative sampling of beneficiaries, to capture the health status, health care use and expenditures of the Medicare population as a whole. The Medicare population differs from the general US population in that it is comprised mainly of people ages 65 and older 84.6 % in 2010) and individuals younger than age 65 with a long term disability (15.4 %). In general, in order to be eligible for Medicare, one must be eligible for either Social Security retirement or disability benefits. A small fraction (<1 %) of Medicare beneficiaries are eligible because of having end-stage renal disease requiring dialysis or transplant or amyotrophic lateral sclerosis.

The MCBS provides a valuable opportunity to evaluate the benefit of using health survey and claims information to evaluate the prevalence of neuropsychiatric disorders. Although the MCBS is commonly used for cost estimates of the entire population, its unique combination of survey and claims data represents an ideal resource for constructing a method that uses multiple sources of data to identify those with potentially cognitively impairing neuropsychiatric conditions. Although it does not contain a “gold standard” for measuring these conditions, it allows examination of the contributions and potential limitations of each source, and can demonstrate how estimates of the proportion of the population affected by these conditions may vary depending on the data sources used.

The goal of our study was to develop a method that combines various sources of information available in the MCBS to identify people with diagnoses of intellectual/developmental disabilities, dementia, mental illness and neurological conditions affecting the brain. Since most studies are done either using claims only or survey only, we wished to compare the differences in the prevalence of these disorders using health survey information only, claims only and health survey plus claims information. We performed this comparison in the community sample, long-term care facility sample and overall sample.

## Methods

### Medicare Current Beneficiary Survey

The United States Medicare Current Beneficiary Survey (MCBS) uses a stratified multistage probability sample design to draw a nationally representative sample of Medicare beneficiaries. The first sampling stage selects 107 nationally representative primary sampling units (PSUs) which consist of counties (or multiple counties) with both metropolitan and non-metropolitan areas. The second stage selects zip code clusters within each PSU. The third stage, beneficiaries in the selected zip codes are stratified by seven age groups (under age 45, 45–64, 65–69, 70–74, 75–79, 80–84, and 85 and older) and then subsampled at rates designed to provide equal probability samples within each of the seven age groups [6]. Younger beneficiaries (under age 65) and the oldest beneficiaries (over age 85) are oversampled to improve estimates for these vulnerable segments of the population [7, 8]. The relative sampling rates can range from a low of 1 (70–74) to a high of almost 4 (under age 45 group) [9]. Every year a new panel is selected, and each panel remains in the survey for four years. Participation is not mandatory and initial response rates are about 80 % with follow-up participation rates around 95 %.

We used the 2010 Access to Care sample, which consists of Medicare beneficiaries who were enrolled as of January 1, 2010 and remained enrolled the entire year. It includes four panels (*n* = 14,672), the panels entering in 2007, 2008, 2009 and 2010. Their cumulative response rates for the 2010 fall survey were 56.6, 58.9, 61.2 and 77.5 % respectively [10]. The MCBS adjusts its survey weights to account for non-response to reduce potential non response bias [11]. The MCBS divides respondents into the community-dwelling and the long-term care facility sample and tailors the survey so that relevant questions are asked of each sample. The MCBS defines long-term care facilities as facilities with at least three beds which provide “long-term care services throughout the facility or in a separately identifiable unit” [10]. Long-term care facilities include long-term nursing homes, assisted living and retirement communities that provide personal care, psychiatric care facilities, and institutions for persons with intellectual or developmental disorders and adult group homes.

The MCBS fall health status and functioning questionnaire has a community version and a long-term care facility version. The questions and structure of the community and facility surveys are different although the topics are similar. The community survey asks respondents (or their proxy) directly about health diagnoses, using the language, “Has a doctor ever told you [or specified person if asking proxy] that (you/he/she) had [specific diagnosis]?” It also asks which diagnoses were the primary causes of Medicare eligibility for those who qualified for Medicare initially because of a disability rather than age (primarily beneficiaries under age 65). Approximately 10 % of community respondents are interviewed using a proxy. In contrast, no facility respondent is interviewed directly; instead a facility staff member serves as the proxy. Since nursing homes certified by the Centers for Medicare and Medicaid Services are required to perform a clinical assessment of their patients and fill out a Minimum Data Set (MDS) form on each patient, the facility staff member is instructed to refer primarily to a participant’s MDS quarterly review and if necessary their full MDS assessment for answers to the MCBS questions. In cases where an MDS assessment is not available, the staff proxy is instructed to refer to the participant’s medical chart [10].

For participants in a long-term care facility, the MCBS surveys the facility in which they reside about its characteristics and provides that information in the MCBS files. The MCBS files also contain information from Medicare administrative records for all participants. Medicare claims files of participants are provided with the 2010 Access to Care MCBS files. We limited our analysis to Fee-For-Service (FFS) beneficiaries excluding those who were in an HMO (*n* = 3894) because Medicare HMOs are not required to submit claims. We used all available claim files: inpatient hospital, outpatient hospital, skilled nursing facility, physician/supplier, home health, hospice, and durable medical equipment claims. The Cost and Use dataset contains a file with nursing home MDS assessments of participants in the continuing panels who received an assessment during the year, but not for the entering panel. Information from the MDS file and Medicare claims files can be matched to the Access to Care participants using the participant identifier.

### Neuropsychiatric conditions

The strength of the MCBS is its combination of survey data, administrative data and claims. We wanted to take advantage of these various sources of information to do a broad screen for neurological, cognitive, and psychiatric conditions that may cause or are often associated with cognitive or mental impairment. We classified conditions according to four broad, but clinically distinct categories: intellectual/developmental disorders (ID/DD), neurological conditions affecting the central nervous system (Neuro-CNS), dementia and psychiatric conditions (Table 1). For each of the 4 broad categories used in building our classification system we developed an approach that captured and combined information from two sources: claims and the MCBS health status and functioning community and facility surveys. Table 1 contains the diagnoses used in each category (column 1), the International Classification of Diseases – 9 (ICD-9) codes used to identify the various diagnoses (column 2), and the health status and functioning survey variables which were used in the community (column 3) and facility (column 4) surveys. ICD-9 codes consist of 3–5 numbers. The first three numbers specify a category, the forth digit specifies the subcategory and the fifth digit specifies the sub-classification. In Table 1, we use 3 digits when we used all subcategories and sub-classifications of a particular category, 4 digits when we used all sub-classifications of a particular subcategory and 5 digits when we only used a particular sub-classification.

**Table 1:** Medicare Current Beneficiary Survey (MCBS) Variable Names and ICD-9 codes used in classification system

Diagnostic Category and Sub-Diagnoses	ICD-9 Codes	Community Health Survey (HS) Variables	Facility HS Variables
Intellectual and Developmental Disorders (ID/DD)^*^			
Intellectual Disability	317, 318x, 319, V792	ocmental, emcausc1/emcausc2 = 22^**^	n/a
Cerebral Palsy	343x	emcausc1/emcausc2 = 9	cerpalsy
Delay/Pervasive DD	3141, 315xx, 299xx, V93, V98, V99	none	none
Spina Bifida	741x	none	none
Chromosomal	7580-3, 7586-9, 7595, 7598	none	none
Brain: Anomalies, Degenerative	330, 740, 742, 7560, 75555	none	none
Muscular Dystrophy	3590-2	emcausc1/emcausc2 = 8	none
Congenital Infections	7710-2	none	none
Endocrine, Toxic	243, 2530, 7607, 33181	none	none
Metabolic	270, 2772, 2775, 27781-2, 27785-9	none	none
Psychiatric Disorders			
Schizophrenia/Delusion	295, 297, 2980, 2984, 2989	none	schizoph
Bipolar	2960-1, 2964-9	none	manicdep
Depression/Mood	2962-3, 3004, 311,v790,	ocdeprss, emcausc1/ emcausc2 = 34	depress
Personality	301xx	none	none
Anxiety/Somatic/Dissociative	3000x, 30011-30015, 30021-30023, 3003-3007, 3008x, 3009, 30981	none	anxiety
Eating Disorders	3071, 3075x	none	none
ADHD/Conduct/impulse Control	314xx, 312xx	none	none
Unspecified	n/a	ocpsycho, emscausc1/2 = 35	n/a
Neurological (Central Nervous System)			
Traumatic Brain Injury	850-4, 8001-4, 8006-9, 8021-4, 8026-9, 8031-4, 8036-9, 8041-4, 8046-9, 95901, 9070, 3102, 2930, 2940	none	braininj
Stroke	430, 431, 432x, 433xx, 434xx, 437x, 438xx	ocstroke, emcausc1/emcausc2 = 16	stroke
Epilepsy	3450-5, 3457-9	emcausc1/emcausc2 = 5	seizure
Aphasia	7843	none	aphasia
Brain: Hydrocephalus, Degenerative	3313-4, 33183, 33189, 3319, 3481	none	none
Multiple Sclerosis/Demyelinating	340x, 3411, 3418, 3419	emcausc1/emcausc2 = 7	scleros
Parkinson/Movement	332, 333.0, 333.4	ocparkin, emcausc1/emcausc2 = 27	parknson
Brain Cancer	191, 1920-1, 1983	occbrain	none
Dementia			
Alzheimer's	3310	no specific	alzhmr
Unspecified	290, 2912, 29282, 2941, 2948, 797, 3311, 3312, 33182	ocalzhmr, emcausc1/emcausc2 = 23	dement

^*^ID/DD Diagnostic Category also used the Minimum Data Set file (ric.mds) variables (ab10a = 0, ab5E = 1); and the facility interview file (ric7) variable "plactype" = 16

^**^emcausc1 and emcausc2 are the variable names which list the diagnosis (coded as a number) that was the cause of Medicare eligibility for people under 65 at the time of their initial MCBS interview. Only one of the two had to list the diagnosis to be included

### Intellectual and other developmental disabilities

#### Claims definition

In addition to ICD-9 codes for intellectual disorder, we chose developmental disorders (and their related ICD-9) codes after a review of the literature relying heavily on the list of conditions associated with intellectual disorder used in the analysis of the 1994/1995 National Health Interview Survey Disability Supplements [12, 13]. We used ICD-9 codes for the following developmental disorders: intellectual disorder, cerebral palsy, developmental delay, spina bifida (we excluded spina bifida occulta), deformities of brain and skull, chromosomal abnormalities, muscular dystrophy, congenital infections, congenital endocrine disorders such as congenital hypothyroidism and acromegaly, metabolic disorders (Table 1).

#### Health status and functioning questionnaires

The community health status questionnaire asks “Has a doctor ever told (you/SP) that (you/he/she) had mental retardation?” Those under age 65 were asked an additional question, which provided mental retardation as a possible answer: “Which of these conditions was the cause of (your/SP’s) becoming eligible for Medicare?” A positive answer to either of these questions was counted as an intellectual disorder. There were no questions specifically asking about other developmental disabilities, but muscular dystrophy and cerebral palsy were among the listed causes of Medicare eligibility and those who had these conditions listed were considered to have a developmental disorder.

The facility health status survey combines ID/DD and mental illness into one question. “Did (SP)'s record indicate any history of mental retardation, mental illness, or developmental disability problems? Exclude diagnoses of organic brain syndrome, Alzheimer's disease, and related dementia.” Thus it is not possible to separate out those with an ID/DD purely from the facility health status interview. The survey does contain a specific question on cerebral palsy which was included in the survey definition of ID/DD. Because of this limitation in the MCBS survey, for this category alone, we supplemented the health survey information with information from the MDS file and facility questionnaire. We used the MDS files provided with the Cost and Use data set, which has a question (question ab10a) that allows the identification of ID/DD status. Facilities fill out a survey describing institutional characteristics including type of facility. Participants in a facility that responded that it was an “institution for the mentally retarded/developmentally disabled” were considered to have an ID/DD. The MDS file provided also contains questions on residential history in the prior 5 years (question ab5). We considered participants to have ID/DD if it was indicated in question ab10a or if they had a history of residential stay in an ID/DD facility.

### Psychiatric disorders

#### Claims definition

Our goal was to include claims that were evidence for a chronic psychiatric disorder [14], thus we excluded codes for acute disorders such as acute psychoses, and adjustment disorders. We also excluded simple phobias. We did not include codes purely related to alcohol and substance abuse disorders. The subcategories of psychiatric disorders included are listed in Table 1.

#### Health status and functioning questionnaires

The community health questionnaire asks whether a doctor has ever told a participant that s/he has depression or a mental or psychiatric disorder other than depression. Participants in an entering panel who are under age 65 are asked whether depression or a mental disorder other than depression was the cause of Medicare eligibility.

We did not use the facility health status and functioning survey’s general question asking about mental disorders combined with mental retardation/developmental disorders. The facility health questionnaire did ask about the following specific psychiatric disorders: anxiety, bipolar disorder, depression, and schizophrenia, which were used in our case definition.

### Neurological disorders affecting central nervous system

#### Claims

For claims we used the ICD-9 codes associated with all of the disorders asked about in the community and facility health surveys (Table 1). In addition we added diagnostic codes for hydrocephalus, encephalopathy and anoxic brain damage. We coded Parkinson’s disease using codes for primary (332.1) and secondary (332.2) Parkinson’s as well as the code for other degenerative disorders of the basal ganglia (333.0), but not code 333.1 (essential tremor), which has been shown to have low specificity for Parkinson’s disease [15, 16]. We also used the code for Huntington’s disease (333.4).

#### Health status and functioning questionnaires

The community health status and functioning questionnaire surveys participants about the diagnoses of stroke, Parkinson’s and brain cancer (including metastases). In addition to stroke and Parkinson’s disease, whether people were eligible for Medicare because of seizure disorders or multiple sclerosis is also recorded. The facility questionnaire has staff report on active diagnoses of brain injury, stroke, seizure disorder, aphasia, multiple sclerosis and Parkinson’s disease.

### Dementia

#### Claims definition

Prior research has shown that ICD-9 codes are not very sensitive in distinguishing between Alzheimer’s disease and non-Alzheimer dementia, but do better when dementia as a broad category (including both types) is used [17]. Therefore we combined the diagnostic codes for Alzheimer’s disease and other dementias/cognitive impairment into one category [18] (Table 1).

#### Health status and functioning questionnaires

The community health status questionnaire does not distinguish between Alzheimer’s disease and other dementias, asking “Has a doctor (ever) told (you/SP) that (you/he/she) had Alzheimer’s disease or dementia”. In contrast, the facility questionnaire directs the facility proxy to mark off active diseases on the MDS assessment and both Alzheimer’s disease and dementia (other than Alzheimer’s) are listed as options.

### Other health conditions

For the sake of comparison and for further evaluation of the validity of our method, we also included two common non-neuropsychiatric chronic health conditions arthritis and diabetes. Diabetes, a condition that can be asymptomatic, is a condition which we expected to be captured well in administrative data given the frequent use of clinical testing. Arthritis, in contrast, is a condition often identified because of subjective pain complaints which may be better captured with survey data. Both are common in older adults.

We used the National Arthritis Data groups ICD-9 codes for arthritis and other rheumatic conditions which have been used in other studies to estimate population-based prevalence [19, 20]. The community survey asks about rheumatoid arthritis and non-rheumatoid arthritis, which are also listed in the causes of Medicare eligibility questions. The facility survey asks about arthritis without distinction of type.

For diabetes, we used the following claims codes to identify those with a diabetes-related claim: 250xx, 3620x, 3572, 36641. The community health status and functioning questionnaire asks about diabetes and the subtype, and we did not count those who only reported gestational or pre-diabetes on the survey as having diabetes. The facility survey asks about diabetes and diabetic retinopathy and a positive answer to either of those was considered evidence of diabetes.

### Functional status

Activities of daily living (ADL) limitations were expressed as five partially hierarchical and mutually exclusive stages ranging from no difficulty in any ADLs (Stage 0) to difficulty with all ADLs (Stage IV). The initial validation studies of ADL stages were performed with the Longitudinal Study of Aging II sample [21–23] and were re-derived in the MCBS community sample [24]. Community participants were asked: “Because of a health or physical problem, (do you/participant if proxy interview) have any difficulty [by (yourself/himself/herself) and without special equipment] with the following: bathing or showering, dressing, eating, getting in or out of bed or chairs, walking, and using the toilet.” Those who reported having difficulty or who did not do the activity because of a health problem were considered to have difficulty.

The phrasing for facility ADL questions was somewhat different since the facility questions asked about the degree of assistance required for performing each ADL. We assigned those in facilities who were reported to be independent with the ADL without the need of an assistive device as having no difficulty. Those who needed supervision, any level of assistance or did not do the activity were considered to have difficulty.

Questions about the instrumental activities of daily living (IADLs) are similar in structure to the community ADL questions in both community and facility questionnaires. We used the questions about managing money and using the telephone, and assigned difficulty to those who reported difficulty or who did not perform these activities because of a health or physical problem.

### Demographic variables

Demographic covariates including age, sex, race/ethnicity, marital status and education and presence of a living child were obtained primarily from survey data. Where there was missing data we used other sources. For race/ethnicity, we used Medicare administrative data. For income we filled in with income reported in the 2010 Cost and Use files. We did the same for marital status and presence of living child, but we also filled in missing data from household composition, and helper relationship and proxy relationship data. For example if marital status was missing, but person lived with a spouse, received help from a spouse, or the spouse was a proxy, we counted them as married. Administrative data was also used to determine whether they lived in a metropolitan area. For dual eligible status, and participation in an HMO we used variables, which combined Medicare administrative and survey data. Facility living status was determined by whether they received a facility fall 2010 health status and functioning interview and proxy status for those in the community was noted.

### Analysis

Data analysis was performed with SAS® 9.4 software (SAS Institute, Inc., Cary, NC, 2013) and we used the survey procedures to account for the complex survey design. We used the MCBS Access to Care cross-sectional weights in all analyses. These weights enable the production of estimates from the sample that are generalizable to the Medicare population. They also enable correction for differential selection probabilities, non-response, and post stratification adjustments [11, 25]. The 2010 Access to Care cross-sectional weights enable calculation of weighted estimates which are representative of the continuously enrolled (from Jan 1 to Dec 31, 2010) Medicare population. All percentages presented incorporate the survey weights and are thus weighted percentages.

To assess the effect of adding MCBS survey information we calculated sample proportions based on health survey only, claims data only, and both sources The ID/DD category also uses information from the facility and MDS files and these were considered part of “survey” data for this diagnostic category alone. We used descriptive statistics to compare demographic and functional characteristics of those identified with a neuropsychiatric disorder using claims and survey and those without. We compared the demographic characteristics of the two groups as part of evaluating construct validity. We expected that the neuropsychiatric group would have a higher proportion of people in the youngest and oldest groups, a higher prevalence of persons reporting low income, ADL and IADL dysfunction, not being married, being dual eligible, living in a facility and using a proxy.

Diagnosis proportions were calculated for the community, facility and entire (community and facility together) samples. Differences between the proportion of the sample identified using health survey data only, and the proportions using health survey plus claims were calculated in two ways. One was the absolute difference (delta), obtained from subtracting the health survey proportion from the health survey plus claims proportion:

$$ \mathrm{Delta}\ \left(\updelta \right) = \mathrm{Health}\ \mathrm{Survey}\ \&\ \mathrm{claims}\ \mathrm{proportion}\ \hbox{--}\ \mathrm{health}\ \mathrm{survey}\ \mathrm{only}\ \mathrm{proportion}\ \left(\mathrm{absolute}\ \mathrm{difference}\right) $$

The other was the relative percent increase from the health survey only proportion which was calculated as:

$$ \mathrm{Relative}\ \%\ \mathrm{increase} = \left(\left(\mathrm{Health}\ \mathrm{Survey} + \mathrm{Claims}\ \mathrm{proportion}\right)/\left(\mathrm{Survey}\ \mathrm{only}\ \mathrm{proportion}\right)\ \hbox{--}\ 1\right)\ *100\ \% $$

In addition to the impact on estimated proportion of the sample with a diagnosis, we also wished to examine the overlap between cases identified through claims and those identified through health survey. We expected there to be cases identified by claims that were not identified by health surveys because we used major sub-diagnoses of each category regardless of whether there was a question about the diagnosis on the community and facility health surveys. To gain a better understanding of the agreement between sources, we chose individual sub-diagnoses of the psychiatric (depression) and Neuro-CNS (stroke, Parkinson’s) categories that had a specific question about the diagnosis in the facility health survey, and the community health survey as well as specific diagnostic codes. We used the total number of cases identified using claims plus health survey information and calculated what percentage were identified through claims alone, health survey alone and both.

## Results

The overall estimated prevalence of neuropsychiatric disorders using health survey plus claims was 50.0 %. The distribution of demographic and functional characteristics of those with and without neuropsychiatric disorders followed expected patterns (Table 2). A much greater percentage of those with neuropsychiatric disorders (10.0 %) lived in a long-term care facility compared to 0.9 % of those without such a disorder. A greater proportion of those with neuropsychiatric disorders were under age 65, female, African American, not married, had low income and dual eligible status compared to those without neuropsychiatric disorders. As expected they had a higher prevalence of difficulty with managing money, using the telephone, and more difficulty with ADLs. Community-dwelling participants with neuropsychiatric disorders had higher rates of proxy usage.

**Table 2:** Demographic and Functional Characteristics of those with and without neuropsychiatric disorders (determined using claims plus MCBS survey data)

Characteristic		Overall (*n* = 10,778)	Neuropsych Disorder (*n* = 5866 (50 %)	No Neuropsych Disorder (*n* = 4912 (50 %))
		raw n (column wt. %)	column wt. %	column wt. %
Age	<65	2163 (17.6)	27.3	7.9
	65–79	5074 (57.2)	44.4	70.0
	80+	3541 (25.2)	28.3	22.1
Sex	Female	5855 (54.3)	57.7	50.8
	Male	4923 (45.7)	42.3	49.2
Race/ Ethnicity	White NH	8453 (79.3)	78.5	80.0
	Black NH	1105 (9.3)	10.0	8.6
	Hispanic	711 (6.4)	6.6	6.2
	Other	509 (5.0)	4.9	5.2
Marital Status (miss: 5)	Married	4791 (49.3)	41.3	57.3
	Not Married	5982 (50.7)	58.7	42.7
Income	<25K	5971 (50.4)	59.7	41.0
	≥25K	4807 (49.6)	40.3	59.0
High School Graduate	No	2795 (23.4)	27.0	19.7
	Yes	7814 (75.4)	71.1	79.7
	Unknown	169 (1.2)	1.9	0.5
In Facility	No	9895 (94.6)	90.0	99.1
	Yes	883 (5.4)	10.0	0.9
Dual Eligible	No	8029 (79.4)	70.7	88.2
	Yes	2749 (20.6)	29.3	11.8
Activities of Daily Living Stage (miss: 36)	0	6557 (64.3)	51.0	77.7
	I	1727 (15.7)	17.8	13.5
	II	960 (8.3)	12.3	4.3
	III	985 (8.3)	12.6	4.0
	IV	513 (3.4)	6.4	0.5
Difficulty Money (miss: 43)	Yes	1901 (13.6)	24.0	3.3
	No	8834 (86.4)	76.0	96.7
Difficulty Phone (miss: 42)	Yes	1338 (10.2)	15.7	4.6
	No	9398 (89.8)	84.3	95.4
Community only				
Proxy	No	8802 (90.6)	86.7	94.1
	Yes	1093 (9.4)	13.3	5.9

*Abbreviations*: *MCBS* Medicare Current Beneficiary Survey, *wt*. weighted, *NH* non-Hispanic, *Neuropsych* neuropsychiatric. Source: Authors' analysis using the Medicare Current Beneficiary Survey, 2010

Although the health survey proportion (38.9 %) and the claims only proportion (33.2 %) were not that different (because of incomplete overlap), using both sources of data greatly increased the estimated sample proportion (50.0 %) which is a 28.6 % relative increase compared to using health survey alone. The magnitude of the increase varied by diagnostic category and setting (Table 3). For example, the estimated prevalence of ID/DD using health survey alone was 2.8 % for the entire sample (community and facility) Adding claims to the health survey information resulted in an estimated prevalence of 4.1 %. Because of the relatively low prevalence of ID/DD disorders in the population, while the absolute difference in prevalence was small (1.3 %), the relative increase was 45.2 %. The largest relative increase was seen with Dementia (91.6 %), followed by NeuroCNS disorders (88.2 %). Similarly, among the individual diagnoses, the greatest relative increase (71.3 %) was seen for stroke diagnoses. For depression, the claims proportion was less than half that of the health survey proportion and augmenting health survey data with claims, resulted in the smallest relative increase (12.4 %). In contrast, for diabetes, the claims proportion was higher than the survey proportion (27.4 vs. 20.0) leading to a larger relative increase when augmenting health survey data with claims.

**Table 3:** Estimated Weighted Proportions of Neuropsychiatric Disorders in the Medicare Population by Data Source and the effect of combining sources

Diagnostic Category or Disorder	Sample	Health Survey* Proportion	Claims Proportion	Health Survey & Claims Proportion	delta^all-survey**^(relative % increase)
Any Neuropsychiatric Disorder	Community Only	36.5	30.5	47.6	11.1 (30.3 %)
	Facility Only	79.8	79.4	91.6	11.9 (14.9 %)
	Overall	38.9	33.2	50.0	11.1 (28.6 %)
Neuropsychiatric Diagnoses Categories					
Intellectual/ Developmental Disorder	Community Only	2.6	1.5	3.5	0.9 (34.8 %)
	Facility Only	6.2	10.9	13.8	7.6 (121.5 %)
	Overall	2.8	2.0	4.1	1.3 (45.2 %)
Psychiatric Disorders	Community Only	27.4	16.9	32.8	5.4 (19.9 %)
	Facility Only	51.1	48.2	64.4	13.3 (25.9 %)
	Overall	28.7	18.7	34.5	5.9 (20.5 %)
NeuroCNS Disorders	Community Only	12.2	16.0	22.8	10.7 (87.4 %)
	Facility Only	27.5	46.4	53.4	25.8 (93.8 %)
	Overall	13.0	17.7	24.5	11.5 (88.2 %)
Dementia	Community Only	2.0	4.3	5.0	3 (147.6 %)
	Facility Only	40.4	48.0	57.7	17.2 (42.6 %)
	Overall	4.1	6.7	7.9	3.8 (91.6 %)
Individual Neuropsychiatric Disorders					
Depression	Community Only	25.7	10.6	28.6	2.9 (11.2 %)
	Facility Only	39.8	33.8	50.2	10.5 (26.3 %)
	Overall	26.5	11.8	29.8	3.3 (12.4 %)
Stroke	Community Only	10.6	11.0	17.7	7.1 (67.3 %)
	Facility Only	12.3	23.5	28.4	16.2 (131.9 %)
	Overall	10.7	11.7	18.3	7.6 (71.3 %)
Parkinson's Disease	Community Only	1.0	1.0	1.4	0.4 (37.1 %)
	Facility Only	5.3	9.0	9.9	4.6 (87.4 %)
	Overall	1.2	1.4	1.8	0.6 (48.9 %)
Non-Neuropsychiatric Disorders					
Diabetes	Community Only	19.7	27.0	29.5	9.8 (49.4 %)
	Facility Only	24.9	34.7	37.3	12.4 (49.8 %)
	Overall	20.0	27.4	29.9	9.9 (49.4 %)
Arthritis	Community Only	56.3	48.4	70.5	14.2 (25.2 %)
	Facility Only	19.3	62.7	67.6	48.3 (249.6 %)
	Overall	54.3	49.2	70.4	16 (29.5 %)

*For ID/DD and the overall neuropsychiatric categories, the facility "Health Survey" prevalence contains ID/DD cases identified through the MDS (panels 2007, 2008, 2009) and facility interview files (all panels) in addition to those identified in the facility health status and functioning file

**Delta = difference between all sources prevalence and health survey only prevalence; Relative % is the percent increase in prevalence from adding claims (delta/health survey prevalence)

Delta may not exactly equal difference of presented numbers because of rounding error

Source: Authors' analysis using the Medicare Current Beneficiary Survey, 2010

Strikingly the largest relative increases in diagnosis proportion were seen for the facility sample. With the exception of dementia, the relative increases were greater than those seen in the community sample. Intellectual/developmental disorders had the largest relative increase (121.5 %) among the four broad neuropsychiatric disorder categories and stroke had the largest relative increase (131.9 %) among the individual neuropsychiatric diagnoses. In contrast to its relatively low relative increase in the community sample (25.2 %), the arthritis proportion had the largest relative increase (249.6 %) in the facility sample (Table 3).

**Fig. 1:**
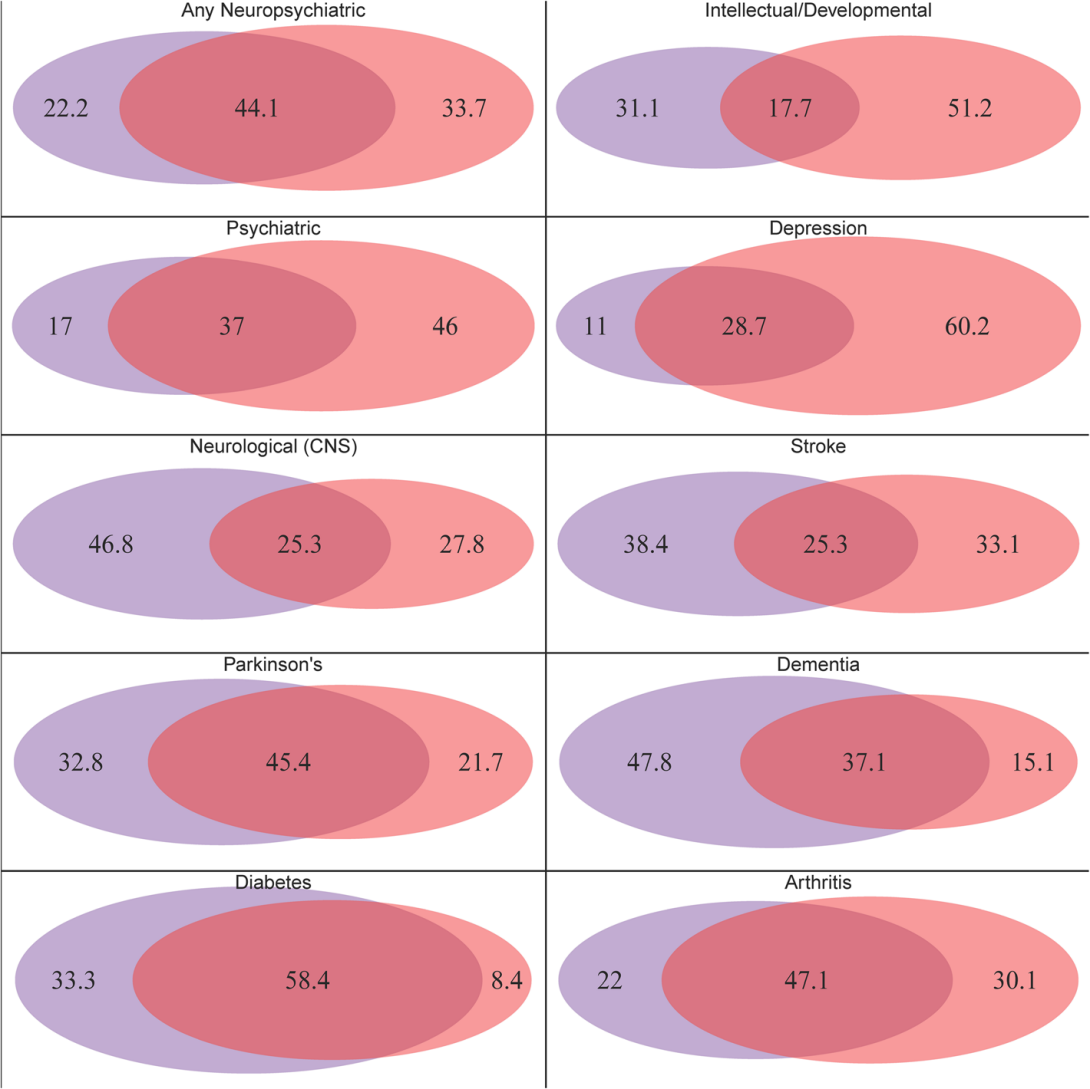
Percent of total cases determined by claims (*purple*), health survey (*orange*) and both (*magenta*) Source: Authors’ analysis of the Medicare Current Beneficiary Survey, 2010

Only 44.1 % of potential neuropsychiatric disorder cases were identified by both claims and health survey (Fig. 1). The lowest overlap was seen in the ID/DD category where only 17.7 % of cases were identified by both and substantially more cases were identified by health survey alone (51.2 %) than by claims alone (31.1 %). The highest overlap was seen in diabetes where 58.4 % of cases were identified by both, 33.3 % by claims alone, and only 8.4 % of cases were identified by health survey alone.

## Discussion

Integrating information from various sources in the MCBS data, most notably health status and functioning survey data with administrative claims increased the identification of people with neuropsychiatric disorders. The estimated proportion of neuropsychiatric disorders in the sample using all sources of information varied as one would expect with demographic and functional characteristics, providing some evidence for construct validity. When health survey information was augmented with claims data the estimated neuropsychiatric disorder prevalence increased by almost 30 %. Among the four individual neuropsychiatric categories, the increase was most notable for respondents with Neuro-CNS disorders followed by intellectual/developmental disorders.

Surprisingly the increases were greatest for those living in facilities, where the staff member proxy relied on the MDS primarily and the medical chart secondarily, raising the question of the adequacy of MDS assessments. We expected more concordance with claims which are also based on the chart. For facilities, the claims proportion was much closer to the combined proportion than the health survey proportion was for all categories and disorders except depression.

In addition, even when the health survey only estimated proportion and claims only estimated proportion did not differ greatly from each other, the increases seen when using both sources suggests each source is capturing different groups of people. Although some studies use surveys as the “gold standard” our findings highlight that this can be inappropriate for neuropsychiatric disorders which can impair a person’s self-awareness or memory, thus impairing the ability to report [26]. Using survey information alone for these diagnoses is likely to lead to underestimates of the proportion of the population affected. Therefore, particularly for those disorders associated with greater cognitive impairments, it appears to be important to augment survey data with claims data. Claims data may also be an important source of augmentation when using interviews done by proxies who may lack full knowledge of the person’s health history. For those living in facilities in particular, claims data contributed to a larger relative increase in diagnosis proportion than the increase seen in community-dwellers with the exception of dementia. It may be especially important to augment facility survey data (based on MDS assessments) with claims data.

On the other hand, claims are notorious for lacking sensitivity for identifying certain disorders [27] and are often not appropriate as a sole source of identification. A person with a neuropsychiatric disorder who sees a physician for other reasons may not have this disorder coded in claims. In addition, certain providers may not code for psychiatric disorders if they think they will not be reimbursed for this code and if the visit also covered other disorders for which reimbursement is easier. Typically, claims data are more time-limited than survey data and are not as good as survey data in ascertaining lifetime occurrence of a disorder.

Our study suggests that the adequacy of either claims or survey data varies with the type of disorder and setting. For example, diabetes, an ongoing chronic condition typically followed using objective laboratory measures and often requiring frequent encounters (which logically generate claims), had relatively few cases identified by survey alone. In contrast, conditions like intellectual disability, which may not need new medical interventions or assessment (hence few claims), had a large number of cases identified by survey only. However, more severe forms of intellectual or developmental disorders may impair ability to self-report, so these disorders also had a large percentage identified by claims alone. Thus, heterogeneity among individuals with the same or related diagnostic codes—in their ability to self-report on a survey or in their need for follow-up visits that generate claims—can lead to substantial differences in the sensitivity of survey versus claims data for case ascertainment and prevalence estimation. A similar pattern was seen with stroke. On the one hand, stroke, which may consist of one episode followed by recovery, had a higher percentage of cases identified by survey alone than Parkinson’s disease, which usually is chronic and requires ongoing health service encounters, making the latter more easily captured through claims. However, one effect of stroke can be anosognosia which would impair ability to report and thus stroke also had a logically higher percentage of cases identified by claims alone with a much smaller overlap between the two sources of data than did Parkinson’s. For arthritis there was a large dependence on setting where the survey prevalence was higher in the community, in contrast to the facility setting, where the disorder was better identified by claims.

### Limitations

The major limitation of this study is the absence of a “gold standard” with which to compare our combined measure. Use of self-report as a gold standard, as has been done in some studies [28, 29], is not appropriate for conditions that are commonly associated with impaired cognitive function. Even medical records cannot be considered the “gold standard” as it is mainly a comparison of the agreement of professional coder versus physician/researcher assessment of the medical records; it does not allow one to assess the accuracy of the diagnosis in the medical records [30]. Prospective clinician assessments of the individual could be considered a gold standard but are not practical and economically feasible to perform on a large scale. Without a “gold standard,” we are not able to determine our false positive and negative rates. Our findings, however, are reasonably consistent across time in the short term as we obtained consistent results in 2005, 2006 and 2010 data.

In general, claims tend to have much lower sensitivity than specificity [2, 27]. As such the addition of claims may not completely compensate for under-reporting on surveys. While it is difficult to directly compare our estimated sample diagnosis proportions with the prevalence found in national surveys because of differences in age structure and disorder definition, our estimates for dementia are reasonably close to the estimates reported by a study using the 2002 US population-based Health and Retirement Study Aging, Demographics and Memory Study (HRS-ADAMS) data [31]. The HRS-ADAMs study included careful in home assessment, neurocognitive battery and diagnoses were made by expert multidisciplinary consensus panel with and without reference to the medical records of the participant. For the age groups 70-79, our estimate of 5.6 % is close to the HRS-ADAMs estimate for 71–79 year olds of 4.97 (2.61–7.32). Our overall estimate for the 70 and above age group (11.6 %) is somewhat lower than the HRS-ADAMs estimate of 13.93 (11.42–16.44) for the age 71 and above group [31], but still reasonably close considering the different sampling frames, years (2002 vs. 2008) and methodologies. It does not appear that we have excessive false positives for dementia.

For depression our community sample estimate of 28.6 % is substantially higher than that of the National Comorbidity Study-Replication lifetime mood disorder prevalence of 20.8 % [32]. This difference is driven somewhat by the very high rate of depression in our under age 65 sample (58.7 %) which is consistent with high rates of clinically significant depressive symptoms (58.3 %) in the under 65 Medicare population reported in other studies [33]. Our over 65 estimate, however, is still higher than the equivalent estimates in other studies and we cannot exclude false positives. The degree to which false positives or false negatives are a concern can depend on the application. We chose to be more inclusive as is appropriate for a broad screen, thus it is likely we included some false positives. Those more concerned about excluding false positives, however, and willing to sacrifice sensitivity, can make the criteria more stringent by requiring multiple occurrences of codes within claims, as has been done in other studies [29, 34].

In addition, using broader categories as we have done, can help decrease the number of false negatives (and false positives.) Other studies have shown that the false positive rate is higher when trying to identify specific diagnoses like Alzheimer’s disease [13]. Using a more general category such as dementia lowers the false positive rate [13]. Therefore we expect our broader category of neuropsychiatric disorders to have a lower false positive rate than narrower categories.

Finally, the study was performed in a US dataset and a US population-based sample representative of the US Medicare population. Thus, the specific proportions presented and the specifics of the survey data are unlikely to be generalizable to other countries. However the underlying principles of the approach should be able to be applied to other surveys/situations where both types of data are available.

## Conclusions

We have created a broad, four-category screen for distinct neuropsychiatric disorders applicable for population-level studies, which overcomes some of the limitations in using claims data or survey data alone to estimate the proportion of the sample that has been affected by neuropsychiatric disorders. In addition, we have illustrated that it is possible to use a national survey such as the MCBS as a feasible source for surveying adults with developmental disabilities – an understudied population. We provide a detailed methodology to enable others to build on our work as is necessary given updates to ICD codes and survey variables. We illustrate that relying on either claims information alone or self-report alone appears to underestimate the sample proportion of neuropsychiatric disorders. However the magnitude of under-estimation is dependent on the specific category of disorder, the specific diagnosis, and the setting. Using both sources together is generally recommended for tasks or projects that require a more accurate estimate of the proportion of the population affected by such conditions. This is especially true of conditions which may impair an individual’s ability to self-report (such as dementia), or for which ICD codes have low sensitivity (such as depression). While our study focused on the MCBS, it is likely this approach will also be valuable to augment other surveys querying about neuropsychiatric conditions.

The MCBS provides an ideal opportunity to understand the relationship between self- or close proxy-reported disorders and claims-identified disorders and the potential biases of each source. An understanding of this relationship is invaluable given that it is not financially feasible for most population-level studies to do a full neuropsychiatric assessment. Furthermore, because of its innovative approach to collecting cost data, the MCBS can be used to better evaluate how using only one source of information might bias cost estimates for those conditions, which is of great importance for policy makers. Consequently, both survey and claims data as combined from the MCBS will continue to be useful in US population surveillance and health services research.

## Abbreviations

FFS: Fee-for-Service
HIPAA: Health Insurance Portability and Accountability Act
HMO: Health Maintenance Organization
ICD: International Classification of Diseases
ID/DD: Intellectual/Developmental Disorders
MCBS: Medicare Current Beneficiary Survey
MDS: Minimum Data Set
Neuro-CNS: Neurological disorders affecting the central nervous system
